# Additive Effect of rPb27 Immunization and Chemotherapy in Experimental Paracoccidioidomycosis

**DOI:** 10.1371/journal.pone.0017885

**Published:** 2011-03-10

**Authors:** Viviane C. Fernandes, Estefânia M. N. Martins, Jankerle N. Boeloni, Juliana B. Coitinho, Rogéria Serakides, Alfredo M. Goes

**Affiliations:** 1 Departamento de Bioquímica e Imunologia, Instituto de Ciências Biológicas, Universidade Federal de Minas Gerais, Belo Horizonte, Brazil; 2 Departamento de Microbiologia, Instituto de Ciências Biológicas, Universidade Federal de Minas Gerais, Belo Horizonte, Brazil; 3 Departamento de Clínica e Cirurgia Veterinária, Escola de Veterinária da Universidade Federal de Minas Gerais, Universidade Federal de Minas Gerais, Belo Horizonte, Brazil; Louisiana State University, United States of America

## Abstract

Paracoccidioidomycosis, PCM, the major systemic mycosis in Latin America, is caused by the termally dimorphic fungus *Paracoccidioides brasiliensis* and requires extended periods of chemotherapy with a significant frequency of relapsing disease. The search for new alternatives of treatment is necessary. rPb27 is an antigenic protein from *P. brasiliensis* that already showed a significant protective activity as a vaccine for PCM in experimental models. The cDNA of rPb27 was subcloned into a pET-DEST 42 plasmid, expressed in *E. coli* with a his-tag and purified by affinity chromatography. Immunization with this recombinant protein and chemotherapy were used together in an attempt to improve treatment of PCM. For this, BALB/c mice were challenged with pathogenic *P. brasiliensis* strain and after immunized with rPb27, in the presence of *Corynebacterium parvum* and Al(OH)_3_, some groups were also treated with fluconazole. After 40 days of treatment, the combined drug/rPb27 administration controlled PCM in the liver and spleen, with long lasting protection, and largely preserved tissues structures of these organs. Additionally, in the lungs after 40 days of treatment there was a significant reduction in the fungal load and size of lesions. At the same time, the levels of TNF-α were higher than infected-only mice. Moreover, significant levels of anti-rPb27 specific IgG1, IgG2a and IgG2b isotypes were detected in the sera of mice immunized with rPb27 fluconazole treated or not. These results showed an additive protective effect of rPb27 immunization and chemotherapy, suggesting that an rPb27-based vaccine can be used to enhance PCM antifungal treatment.

## Introduction

Paracoccidioidomycosis (PCM) is a systemic granulomatous disease caused by *Paracoccidioides brasiliensis*, a termally dimorphic fungus widespread in Latin America mainly affecting rural workers [1]. PCM is the most prevalent systemic endemic mycosis in South America notably in Brazil, Colombia, Venezuela and Argentina [2]. The fatal acute PCM affects the reticule endothelial system, whereas the chronic PCM affects mainly the lung, which shows a granulomatous inflammation with an inefficient cellular immune response [1], [3].

Antifungal chemotherapy is required to control the disease. The conventional treatment of PCM is based on sulfonamides, amphotericin B and azole derivates. Extended periods of therapy are usually required to warrant a good clinical response and avoid relapses [1]. But, the prolonged time of drugs administration causes frequent self-exclusion of the patient from treatment [1]. The introduction of azoles marked an advance in the treatment of fungal diseases, PCM among them. Azoles act on ergosterol biosynthesis at the C-14-demethylation stage, and the resulting ergosterol depletion and accumulation of 14-methylated sterols interferes with the functions of ergosterol as a membrane component, altering the normal permeability and fluidity of the fungal membrane [2]. Imidazoles (ketoconazole) and triazoles (fluconazole, saperconazole, and itraconazole) have been extensively used for the treatment of PCM and have proven effective for clinical purposes, showing fewer side effects than amphotericin B [1].

The above mentioned antifungal antibiotics have drawbacks such as long time of medication (sulfa derivates and azoles), severe renal side effects (amphotericin B), unresponsiveness of some patients to the treatments (all antifungals) [1], [4], [5], [6], [7], [8] and high cost (azoles, lipid formulations of amphotericin B). Therefore, the search for new and more effective strategies to conventional chemotherapy for *P. brasiliensis* and other fungal pathogenic species, with fewer or no side effects, continues [3].

Because of the search for these new alternatives for PCM treatment, several candidate antigen molecules and its mechanisms of protection against *P. brasiliensis* are being studied. Among these molecules, the recombinant protein rPb27 represents an attractive candidate due to its great potential to control this disease as it was demonstrated in previous work [9], in which this protein showed a significant degree of protection in the lungs, livers and spleens of mice immunized with it and posteriorly challenged with a virulent strain of *P. brasiliensis*. In this same work it was shown that this protein is component of F0 fraction of this fungus. This fraction had already demonstrated protective ability in experimental PCM [10].

The association of imunotherapeutics and antifungal agents to treat PCM has also been investigated. The administration of the peptide P10, a 15-amino acid peptide identified in the glycoprotein Gp43, that have already shown the capacity to elicit the secretion of Th1 type cytokines [11], as an adjuvant to the chemicals used in the PCM therapy, showed an improvement of the therapeutic effectiveness of some antifungal agents [11], [12].

In this work we evaluated the immunotherapeutic potential of rPb27 immunization with or without fluconazole chemotherapy to treat PCM as well as the cytokines profile and IgG isotypes production induced by this combined treatment in experimental PCM using BALB/c mice.

## Results

### Cloning, expression and purification of recombinant rPb27

**Figure 1:**
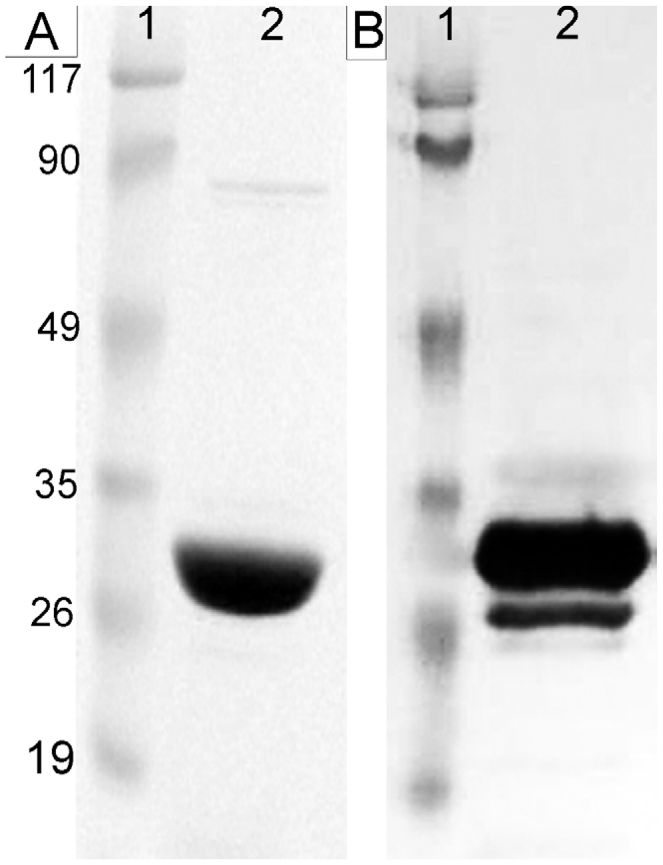
Purified rPb27 profile. **A**. SDS-PAGE analysis of rPb27 after purification on HiTrap™ Chelating HP (Amersham Biosciences, Uppsala Sweden). Aliquot of 20 µl of purified rPb27 was separated on 10% polyacrylamide gels, under reducing conditions, followed by Comassie-blue staining. **B**. Western blotting analysis of purified rPb27 using mouse IgG anti-his-tag (Amersham Biosciences, Uppsala Sweden). Purified rPb27 was subjected to 10% SDS-PAGE, under reducing conditions, followed by eletrophoretic transfer to nitrocellulose paper. The membrane was incubated with mouse IgG anti-his-tag (1∶100) and revealed with goat anti-mouse IgG conjugated with peroxidase (1: 10000). 1, molecular marker. 2, purified rPb27. The molecular weight (kDa) of molecular marker proteins is showed on the left.

Cloning and sequencing of rPb27 DNA resulted in 773 bp comprising the entire rPb27 gene open reading frame and some nucleotides that were added by the plasmid. Nucleotide sequence homology was 100% in relation to the coding region of the 27 kDa *P. brasiliensis* hypothetic protein - access number AA49615 (Data not shown). Nucleotide sequence of this protein was cloned into a pET-DEST42 expression vector where a recombinant protein of approximately 27 kDa was expressed with a his-tag and then purified by affinity cromatography. After this purification it was possible to obtain a single protein with a good yield, as demonstrated by SDS-PAGE and western blotting assays (Fig. 1).

### Organ CFU from intratracheally infected BALB/c mice immunized with rPb27 and/or treated with fluconazole

**Figure 2:**
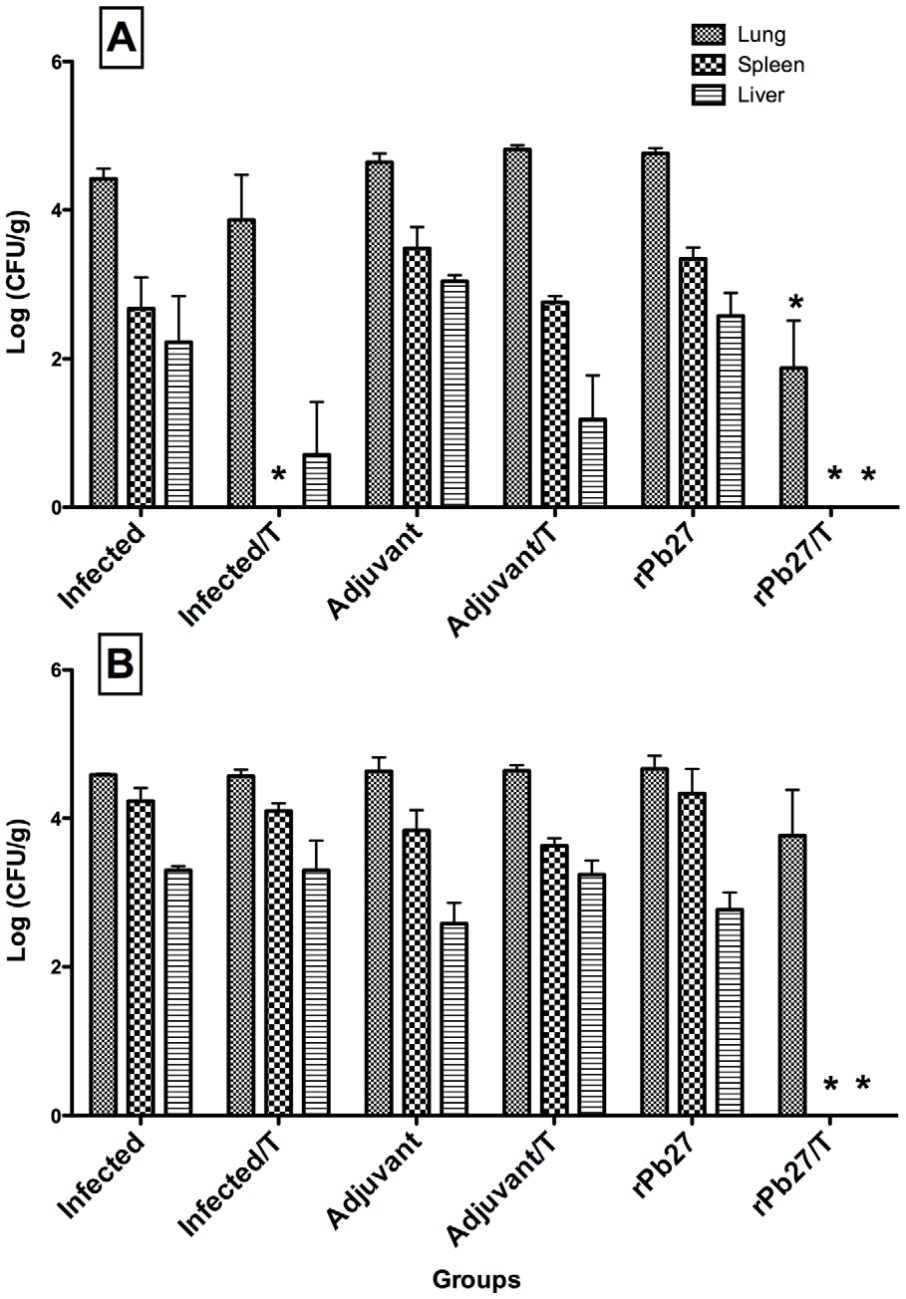
Fungal recovery in lung, spleen and liver of infected mice. The CFUs were estimated 40 (A) and 90 days (B) post treatment in organs from mice infected intratracheally with 3×10^5^ *P. brasiliensis* yeast cells and subjected to fluconazole treatment combined or not with rPb27 immunization. Control mice were only infected with *P. brasiliensis* (Infected), adjuvant mice were inoculated with *C. parvum*-Al(OH)_3_ with fluconazole treatment (Adjuvant/T) or not (Adjuvant), and rPb27 mice were immunized with recombinant protein combined to fluconazole treatment (rPb27/T) or not (rPb27). All groups of mice were infected with the same number of yeast cells. Bars represent the Log_10_(UFC/g) means and standard deviations from organs of 3 to 5 animals in each group. * significant (p<0,05) difference in relation to the group of mice only infected.

To explore the combined effect of rPb27 immunization and fluconazole treatment in BALB/c mice, animals immunization and chemotherapy started 30 days after infection. Analysis of organ CFU was done after 40 and 90 days of treatment. A significant reduction of fungi recovered from lung, spleen and liver of animals (CFU) was obtained in mice immunized with rPb27 and treated with fluconazole at the first time point. In the lung of these animals it was determined, 40 days post treatment, a 60% reduction in the CFUs in relation to infected-only group. Besides, in the liver and spleen of these animals there wasn't recovered any fungi colonie at the two time points analyzed. The rPb27 immunization alone failed to reduce fungi recovered from these organs. And the treatment with the antifungal drug alone also failed to reduce the number of CFU in the lung and liver at two time points, and in the spleen after 90 days of treatment despite a reduction after 40 days (Fig. 2 A, B).

In the lungs despite a significant reduction of fungi recovered at the first time point analyzed in mice immunized with rPb27 and treated with fluconazole (Fig. 2A), after 90 days of treatment this number increased and matched the levels of infected-only group (Fig. 2B).

### Lung, spleen and liver histopathology from BALB/c mice vaccinated with rPb27 and/or treated with fluconazole

**Figure 3:**
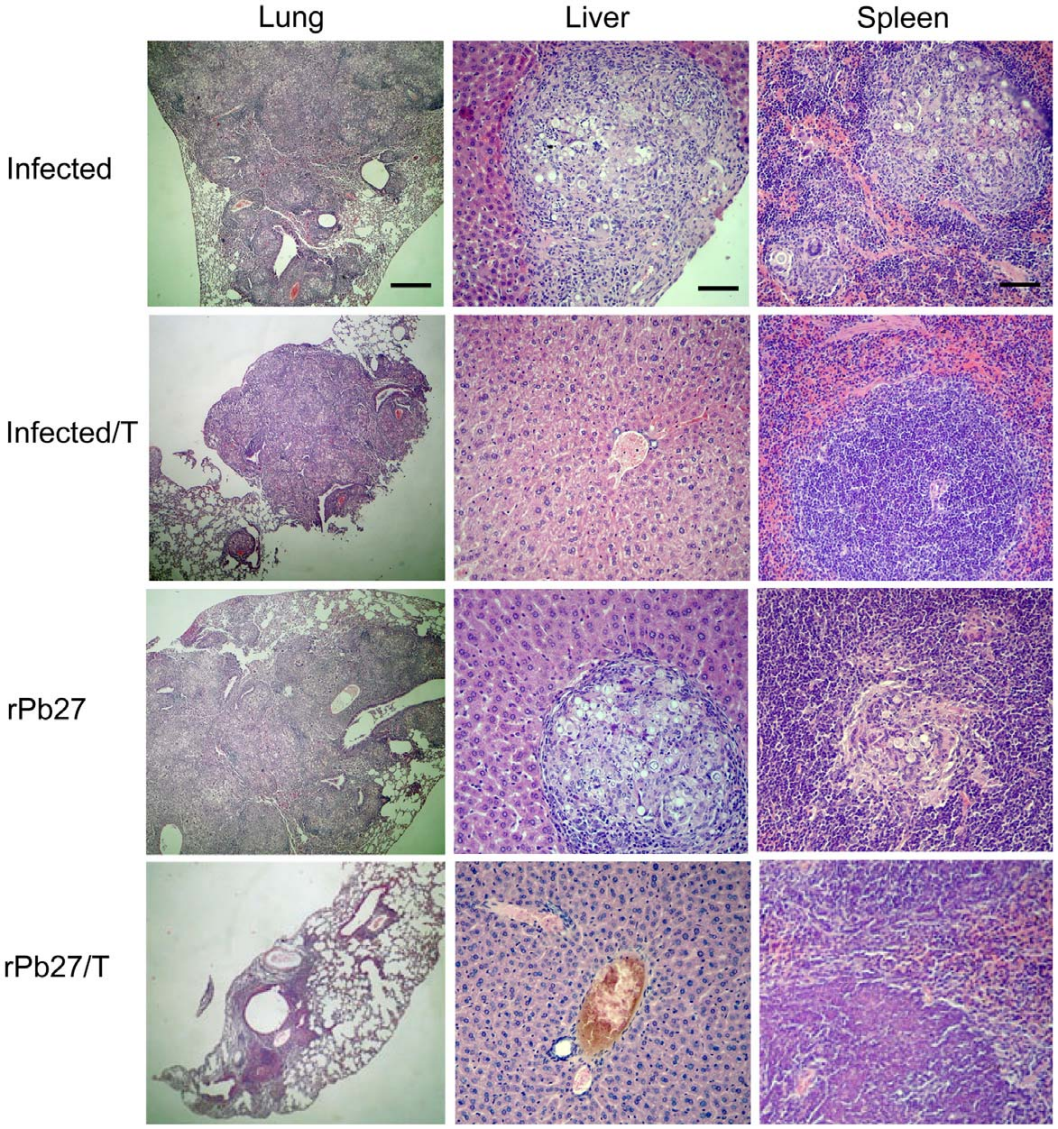
Representative histopathology of lungs, livers and spleens from infected mice, after 40 days of treatment. BALB/c mice were euthanized 40 days after treatment. The lungs, spleens, and livers were excised, fixed in 10% buffered formalin, and embedded in paraffin for sectioning. The sections were stained with hematoxylin–eosin and examined microscopically. Infected, group only infected with *P. brasiliensis*. Infected/T, same as Infected, but treated with fluconazole. rPb27, group infected and posteriorly immunized with rPb27. rPb27/T, same as rPb27, but treated with fluconazole. In each lung photos, the scale bar represents 427.3 µm, while in each liver and spleen photos, the scale bar represents 56.9 µm.

**Figure 4:**
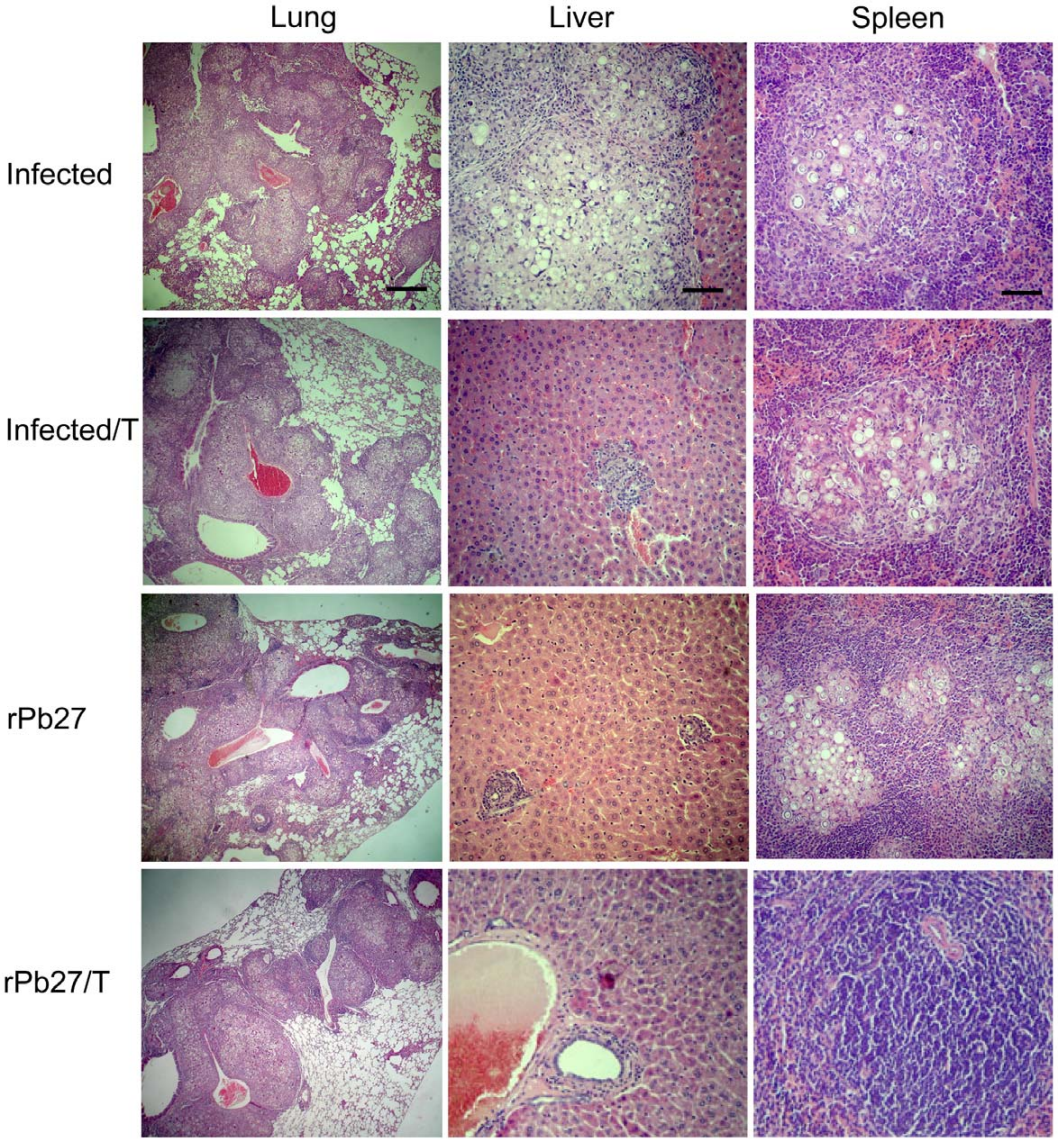
Representative histopathology of lungs, livers and spleens from infected mice, after 90 days of treatment. BALB/c mice were euthanized 90 days after treatment. The lungs, spleens, and livers were excised, fixed in 10% buffered formalin, and embedded in paraffin for sectioning. The sections were stained with hematoxylin–eosin and examined microscopically. Infected, group only infected with *P. brasiliensis*. Infected/T, same as Infected, but treated with fluconazole. rPb27, group infected and posteriorly immunized with rPb27. rPb27/T, same as rPb27, but treated with fluconazole. In each lung photos, the scale bar represents 416.6 µm, while in each liver and spleen photos, the scale bar represents 55.6 µm.

Histopathology of lung, liver and spleen sections showed differences in granuloma lesions. Lungs of animals only infected with *P. brasiliensis*, infected and submitted to fluconazole treatment, or infected and immunized with rPb27 (Fig. 3, 4) showed, at the two time points investigated, giant confluent granulomes with innumerable viable yeast cells of *P. brasiliensis*, and extensive tissue destruction. While in the liver and spleen (Fig. 3, 4) of these groups it was observed multiple foci of granulomatous inflammation, containing fungal cells, however the group infected and treated with fluconazole and the group immunized with rPb27 presented a decrease on lesions size in the liver after 90 days of treatment (Fig. 4) in relation to infected-only group. And at the first time point analyzed (Fig. 3) the group infected and treated with fluconazole didn't present any considerable lesion in the liver and spleen. The groups of mice that were innoculated with adjuvant with or without chemotherapy presented similar pattern of lesions than infected-only mice in the three organs analyzed (data not shown).

**Figure 5:**
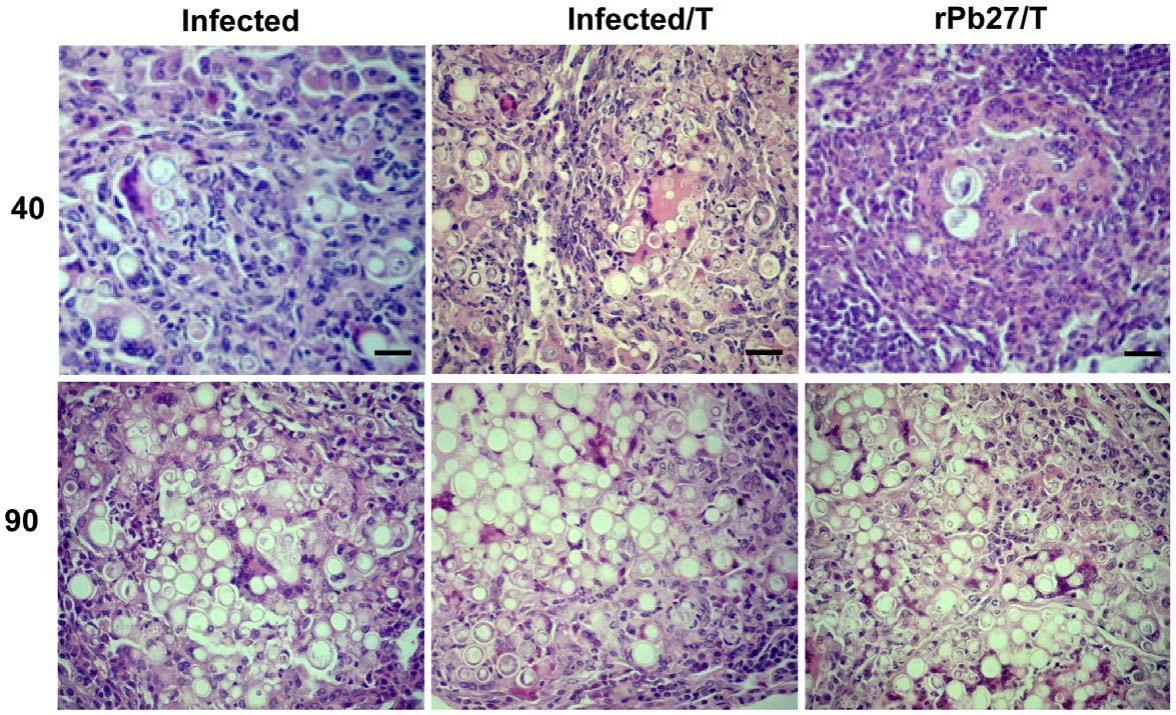
Representative granulome histopathology of lungs from infected mice after 40 and 90 days of infection. BALB/c mice were euthanized 40 and 90 days after treatment. The lungs, spleens, and livers were excised, fixed in 10% buffered formalin, and embedded in paraffin for sectioning. The sections were stained with hematoxylin–eosin and examined microscopically. Infected, group only infected with *P. brasiliensis*. Infected/T, same as Infected, but treated with fluconazole. rPb27/T, group infected and posteriorly immunized with rPb27 and treated with fluconazole. In each photo, the scale bar represents 25.9 µm.

Mice immunized with rPb27 and also treated with fluconazole showed after 40 days of treatment a considerable reduction in the size of lesions at lungs, presenting few pulmonary compact granulomes with no confluence (Fig. 3). These granulomes were more organized and with a reduced number of yeast cells that all other groups analyzed (Fig. 5). The lungs of other infected groups (Infected, Infected/T, Adjuvant, Adjuvant/T and rPb27) presented a desorganized granulome with a great number of viable yeast cells of *P. brasiliensis* (Fig. 5). After 90 days of treatment the group immunized with rPb27 and also treated with fluconazole presented in lungs a pattern of granulome similar to infected group (Fig. 4, 5), and in the spleen and liver this group didn't present any considerable injury at the two time points analyzed (Fig. 3 and 4).

### Recombinant rPb27 specific immunoglobulin responses in infected mice

The specific antibody response to rPb27 in infected mice were evaluated by ELISA. After three rounds of vaccination, significant levels of anti-rPb27 IgG were detected in the sera of mice immunized with rPb27, treated or not with fluconazole. The other groups infected with *P. brasiliensis* didn't present significant antibodie levels for this protein, as well as the group without any intervention (data not shown).

**Figure 6:**
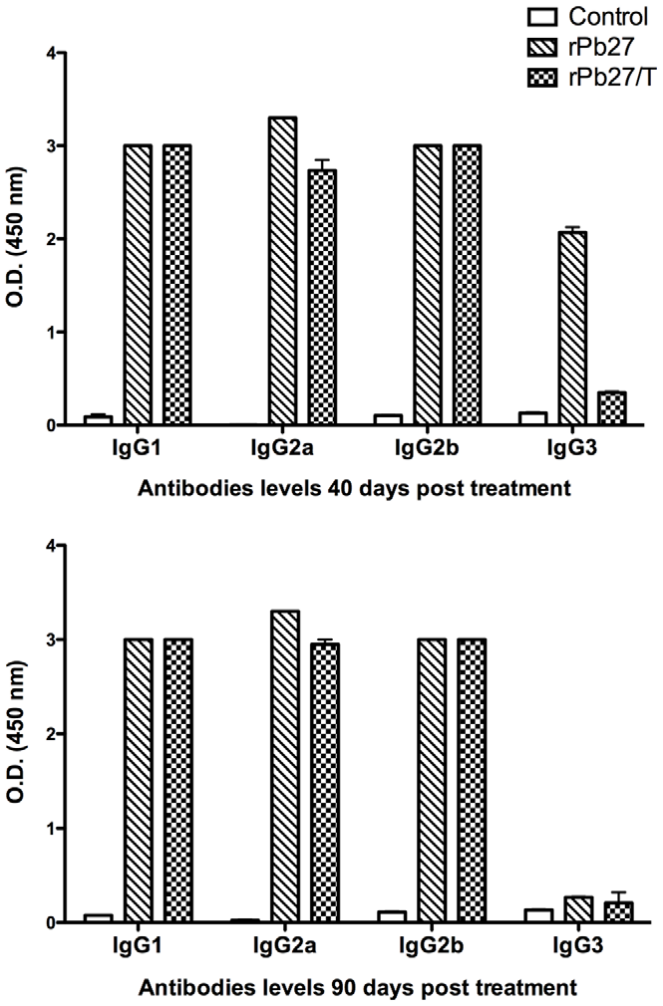
IgG isotypes production against rPb27 by infected and immunized mice. Antibody response against rPb27 in mice infected and immunized with this recombinant protein associated or not with fluconazole chemotherapy was determined by ELISA assay after 40 (A) and 90 (B) days of treatment. Control, mice without any intervention. rPb27, group infected and posteriorly immunized with rPb27. rPb27/T, same as rPb27, but treated with fluconazole. Bars represent the means and standard deviations of optical density (O.D.) at 1∶400 serum dilution in each experimental group (n = 3). * significant (p<0,05) difference in relation to the control group. # significant (p<0,05) difference in relation to the rPb27 group.

Significant levels of anti-rPb27 specific IgG1, IgG2a and IgG2b isotypes were detected in the sera of mice immunized with rPb27 treated or not with fluconazole compared to sera from noninfected mice after 40 (Fig. 6A) and 90 days of treatment (Fig. 6B), while significant levels of IgG3 were found only in the group rPb27 after 40 (Fig. 6A) and 90 days of treatment (Fig. 6B). The other groups infected with *P. brasiliensis* (Infected, Infected/T, Adjuvant, Adjuvant/T) didn't present significant levels of these isotypes (data not shown).

## Discussion

Considering the increase in the worldwide incidence of fungal infections, the development of new therapies for these diseases has become of great importance to public health [13]. The current therapy to treat mycosis is based on polienes and azoles, depending on the severity of the infection [14]. To treat severe cases of PCM amphotericin B followed by itraconazole and sulfametoxazole are indicated. The main disadvantage of using amphotericin B is its occasional toxicity. In the case of itraconazole or sulfametoxazole, the long period of treatment required may cause patients to quit medication, possibly leading to recurrence of disease [14]. Given these dificulties, new approaches to the treatment of systemic fungal infections need to be developed.

Alternative strategies to conventional chemotherapy for fungal diseases have been explored. Combined therapy of immunotherapeutics and antifungal agents in the treatment of PCM has been investigated, and have demonstrated a great potential to enhance antifungal effect and to prevent relapses [11], [12].

Here we reported the efficacy of rPb27 immunization combined with fluconazole chemotherapy in the treatment of PCM. rPb27 is a recombinant protein that was firstly described by McEwen and coworkers [15], and have demonstrated a great potential in immunodiagnosis of PCM [16], [17], [18] and as a vaccine candidate against this mycosis, as demonstrated in previous work, in which the immunization with this protein showed a significant degree of protection in the lungs, livers and spleens of mice posteriorly challenged with a virulent strain of *P. brasiliensis* [9]. In this work the expression and purification of this protein showed a single protein with approximatelly 27 kDa of molecular mass.

After 40 and 90 days of treatment the fungal load was examined in lungs, livers and spleens of infected mice. The pattern of CFU in these organs showed an additive effect of rPb27 immunization and drug treatment. In lungs at the first time point analyzed this combined treatment reduced in 60% the CFU number in relation to control groups (untreated infected animals and those that received adjuvant), however, this CFU number increased after 90 days of treatment reaching the Infected group levels. This relapsing effect in lungs can be assigned to the short time of treatment.

In the liver and spleen the combined treatment reduced in 100% the CFU number in comparison to control groups, at the two time points analyzed. Showing that, in these organs the combined administration of rPb27 and fluconazole controlled the relapsing effects.

Histopathological analysis of infected mice confirmed the therapeutic effect of rPb27 immunization combined with fluconazole chemotherapy, by leading to fungal elimination, reaching sterility in spleen and liver despite the short time of treatment. This sterility wasn't reached by other authors using similar strategies of therapeutic vaccines [11], [12], [19].

In the mice lungs rPb27 immunization with fluconazole chemotherapy reduced CFU in this organ, at the first time point analyzed. In addition, histopathological analyses of the group that received this combined treatment showed less lung tissue compromised 40 days after treatment, presenting large preserved areas with compact, well organized granulomes containing few *P. brasiliensis* yeast cells. But after 90 days of treatment the lungs of this group showed an increase in lung tissue compromised and its granulomes reached the extension and organization of those from control groups, which validates the CFU assay results. Preliminary cytokine screening showed that animals that received rPb27 immunization with fluconazole chemotherapy presented high levels of TNF-α when compared with only-infected group after 40 days of treatment (data not shown). This result conforms with the literature that TNF-α has a protective role in *P. brasiliensis* infection and may have contributed to the reduced size of lung tissue compromised in addition to a decrease on CFU numbers and to a better organization of granulomes when compared to only-infected group. TNF-α is a Th1 cytokine that according to previous work has a great importance to control dissemination and growth of the fungi, and inflammatory response in PCM [20]. These authors used mice with homologous disruption of the TNF-α receptor p55 infected with *P. brasiliensis* and demonstrated that these animals, but not wild-type mice, were unable to control the growth of yeast cells and the mice succumbed to infection by day 90 after infection. Besides, inflammatory granulomas weren't found in these knockout animals. These findings showed that TNF-α mediate resistance to *P. brasiliensis* infection [20]. In fact, the granulomatous inflammatory reaction, a specialized and efficient tissue response against certain parasites [21], *P. brasiliensis* among them, requires TNF-α which is supposed to be responsible for attracting and activating effector cells as well as for macrophage accumulation and differentiation [22]. Besides this, a recent work showed that spleen cells from infected female mice produced more TNF-α than those from males in response to paracoccin stimulus, which contributes to the greater resistance to PCM presented by female in relation to male mice [23].

Summarizing, the rPb27 immunization combined to fluconazole chemotherapy was efficient to avoid fungal dissemination to spleen and liver of mice and to control yeast cells growth in the lungs after 40 days of treatment, but this control in lungs was lost after the suspension of chemotherapy, 90 days post treatment, this can be due to the short time of treatment, only thirty days. In the literature it was mentioned that resistance to *P. brasiliensis* infection is determined mainly by the ability of the host to restrict fungal dissemination rather than control fungal growth at the primary site of infection [24]. This containment of the fungus in the lungs can be the first step to PCM control, and, possibly, with a larger time of chemotherapy this disease could be controlled.

The specific antibody levels was also evaluated. We demonstrated that immunization of BALB/c mice with rPb27 induced significant levels of all isotypes evaluated after 40 and 90 days of treatment. When this immunization was combined with fluconazole chemotherapy mice developed a pulmonary-restricted PCM associated with low mortality rates and production of significant levels of IgG1, IgG2a and IgG2b isotypes after 40 and 90 days of treatment, with a small production of IgG3 only 40 days after treatment. The presented data showed that the immunization with rPb27 promoted enhanced antifungal protection by fluconazole chemotherapy, decreasing fungal load in lungs after 40 days of treatment and avoiding fungal dissemination to other sites of infection (liver and spleen). Thus, rPb27 can be explored as an adjuvant for PCM therapy.

## Materials and Methods

### Ethics Statement

All animals were handled in strict accordance with good animal practice as defined by the relevant national and local animal welfare bodies, and all animal work was approved by the Ethics Committee on Animal Experimentation (CETEA) from the Universidade Federal de Minas Gerais (UFMG). The protocol number is 24/2006.

### Animals used in this work

Adult male BALB/c mice (6–8 weeks old) were purchased from Centro de Bioterismo, ICB-UFMG (Belo Horizonte, MG, Brazil), and maintained under standard laboratory care as previously described [10].

### *P. brasiliensis* strain

Virulent *P. brasiliensis* human isolate was obtained from a patient with active PCM, whose case was reported by Araujo and coworkers [25], maintained in YPD agar medium [0.5% yeast extract, 0.5% peptone, 1.5% D-glucose, 1.5% agar, pH 7.0] (Sigma, St. Louis, MO, USA) at 36°C and collected on the seventh day of culture. The viability of fungal suspensions determined by staining with Janus Green B vital dye method [26] (Merck. Darmstadt, Germany) was always higher than 90%. The virulence of the human isolate was checked in each experiment by infecting intratracheally BALB/c mice and recovering the yeast cells from their organs.

### Subcloning and sequencing of rPb27 DNA

The sequence of the recombinant rPb27 was already cloned by our group in previous work into the expression vector pGEX 4T-2 (GIBCO BRL), which produces a recombinant protein fused to glutathione S-transferase (GST) [9]. In order to facilitate the purification procedure and avoid the contamination of purified protein with thrombin, the rPb27 sequence was transferred to the expression vector pET-DEST 42 (Invitrogen, Carlsbad, USA) which express the recombinant protein with a C-terminal His-tag. The cloning procedure was based on Gateway® technology (Invitrogen, Carlsbad, USA), that uses an strategy based on the recombinational properties of bacteriophage lambda [27]. The primer set for amplification of the rPb27 sequence included the forward primer: 5′- GGGG ACA AGT TTG TAC AAA AAA GCA GGC TTC GAA GGA GAT AGA ATG GCA CGA GCG CTC AGT TC -3′ and reverse primer: 5′- GGG GAC CAC TTT GTA CAA GAA AGC TGG GTC GTT GTG GAA GAC AGC GCT GCA -3′. The PCR annealing temperature was 56°C. The PCR products were separated in 1% agarose gel. The blunt-end PCR products were then cloned into pDONR 221 vector according to the manufacturer's protocol (Invitrogen, USA). The reaction mixture was incubated overnight at 25°C. The reaction was then stopped with 10 minutes Proteinase K incubation. After this ste, competent *E. coli* (TOP10) was transformed by electroporation with the pDONR 221/rPb27 construct according to the manufacturer's protocol (Invitrogen, USA). Positive clones were selected on (LB) medium containing 50 µg/mL kanamycin. Plasmid DNA was isolated using the high pure plasmid extraction kit, Mini-prep QIAprep Spin miniprep 150 (Qiagen, Hilden, Germany). The presence of the insert was confirmed by PCR and, finally, to confirm the fidelity of the sequence, DNA sequencing was performed. This construct is called the entry clone. The LR recombination reaction was then carried out between the entry clone and destination vector, pET-DEST42, according to the manufacturer's instructions (Invitrogen, USA). Competent *E. coli* (BL21) were transformed by electroporation with the products of LR recombination according to the manufacturer's protocol. Positive clones were analysed by culturing them on LB medium containing 100 µg/mL ampicillin. The recombinant plasmid pET-DEST42/rPb27 was checked for accurate insertion by restriction enzyme analysis, and the inserted fragment was sequenced on a MegaBACE DNA Analysis System (Amersham Biosciences, Buckinghamshire, England) [28]. The sequence homology with rPb27 was analyzed using the algorithm ClustalW available on the Internet: http://www.ebi.ac.uk/clustalw/.

### Expression in *E. coli* and purification of rPb27

The recombinant protein, rPb27, was expressed in *E. coli* using the expression vector pET-DEST42 (Invitrogen, USA), which produces a recombinant protein with a C-terminal his-tag. Purification of the recombinant protein was therefore undertaken by HiTrap^TM^ Chelating HP according to the manufacturer's instructions (Amersham Biosciences, Uppsala Sweden).

### Sodium dodecyl sulfate-polyacrylamide gel electrophoresis (SDS-PAGE) and Western blotting

Purified rPb27 was subjected to continuous electrophoresis using SDS 10% polyacrylamide gels under reducing conditions [29]. The separated proteins were stained with Coomassie blue or transferred to a nitrocellulose membrane [30], blocked with 1.6% of casein in phosphatebuffered saline (PBS), pH 7.4, for 1 h at room temperature and then incubated with mouse IgG anti-his-tag (Amersham Biosciences, Uppsala Sweden) diluted 1∶100 in 0.25% casein-PBS for 1 h at room temperature. After this step the membranes were incubated with specific secondary antibody peroxidase-conjugated diluted 1∶10000 in 0.25% casein-PBS for 1 h at room temperature and developed with diaminobenzidine (0.6 mg/ml) in PBS, pH 7.4, plus H_2_O_2_ (1 µl/ml) and NiCl_2_ (10 µl/ml). The reaction was stopped with deionized water.

### Intratracheal infection of BALB/c mice

Male BALB/c mice were inoculated intratracheally with 3×10^5^ viable yeast cells of virulent *P. brasiliensis*, grown on YPD-agar and suspended in sterile PBS. Briefly, mice were anesthetized i.m. with 40 µl of a solution containing 57% of ketamine (Dopalen, Vetbrands, Brazil) and 43% of xylazine (Dopaser, Laboratório Calier do Brasil LTDA, Brazil); after approximately 10 min, their necks were hyper-extended, and the trachea was exposed at the level of the thyroid and injected with 3×10^5^ yeast cells in 50 µl of PBS using a 30-gauge needle. The incisions were sutured with 4-0 silk.

### Immunization of mice with rPb27

After 30 days of infection, groups of male BALB/c mice were immunized by subcutaneous injection of 50 µg rPb27 in the presence of 100 µg *Corynebacterium parvum* and 1 mg aluminum hydroxide, Al(OH)_3_, as an adjuvant. Animals were boosted three times, at two week intervals, with the same amount of antigen. Control mice were inoculated with adjuvant.

### Chemotherapy of infected mice

After 30 days of infection animals were treated for 30 days during which groups of mice received doses every 24 h of fluconazole 10 mg/Kg (Mantena laboratories limited). All drug administration were intraperitoneal.

### Groups of mice studied

Male BALB/c mice were divided in seven groups of 12 animals each: Immunized with rPb27 treated with fluconazole (rPb27/T) or not (rPb27); injected with *C. parvum*-Al(OH)_3_ as an adjuvant fluconazole treated (Adjuvant/T) or not (Adjuvant); infected only (Infected); infected and treated with fluconazole (Infected/T); and a control group without any intervention (Control).

### Fungal recovery in organs of infected mice

Organ colony-forming units (CFUs) were evaluated after 40 and 90 days of treatment in the lungs, spleens and livers, which were removed, weighed and homogenized in PBS. The final suspension was placed on brain heart infusion (BHI, Difco) agar supplemented with 4% fetal calf serum and 5% spent culture medium of *P. brasiliensis* as a growth factor. Gentamycin was added at 40 mg/l. The plates were incubated at 36°C and read after 20 days. The results were expressed as the number of log_10_ of viable *P. brasiliensis* CFUs per gram of tissue per mouse.

### Enzyme-linked immunosorbent assay (ELISA)

ELISA plates were coated overnight at 4°C with 1 µg/100 µl of purified rPb27 recombinant protein in 0.05 M carbonate/bicarbonate buffer, pH 9.6. Wells were blocked for 1 h at 37°C with 1.6% casein in PBS solution. Plates were then incubated with 100 µl of anti-rPb27 mouse serum or non-immunized mouse serum samples diluted 1∶400 in 0.25% casein in PBS for 1 h at 37°C. Washes were performed with PBS-T20 (0.15 M PBS, pH 7.4; 0.1% Tween-20) between incubations. For each well, 100 µl of peroxidase-conjugated goat anti-mouse IgG, IgG1, IgG2a, IgG2b or IgG3 antibodies (Southern Biotechnology Associates, Inc., Birmingham, AL, USA) diluted 1∶5000 were added and incubated for 1 h at 37°C. After additional washes, peroxidase activity was assayed with 100 µl of TMB ELISA substrates solution (Thermo Scientific Pierce). Color development was stopped with 50 µl of 2N H_2_SO_4_. The optical density at 450 nm was measured with an automated ELISA reader (ELX 800 BIO-TEK Instruments Inc.).

### Histopathology of lung, spleen, and liver of experimental groups

BALB/c mice were euthanized 40 and 90 days after treatment. The lungs, spleens, and livers were excised, fixed in 10% buffered formalin, and embedded in paraffin for sectioning. The sections were stained with hematoxylin–eosin and examined microscopically [9].

### Statistical analysis

Data were analyzed statistically by one-way ANOVA followed by the Bonferroni test or Student t-test associated when necessary to the non-parametric Mann–Whitney test, with the level of significance set at p<0.05.
